# The influence of a digital clinical reasoning test on medical student learning behavior during clinical clerkships

**DOI:** 10.1007/s10459-023-10288-x

**Published:** 2023-10-18

**Authors:** Larissa IA Ruczynski, Bas JJW Schouwenberg, Eugène Custers, Cornelia RMG Fluit, Marjolein HJ van de Pol

**Affiliations:** 1 Research on Learning and Education, Radboudumc Health Academy, Radboud University Medical Center, Gerard van Swietenlaan 2 (route 51), 6525 GB, Nijmegen, Netherlands; 2 Department of Pharmacology and Toxicology, Radboud University Medical Center Nijmegen, Nijmegen, the Netherlands; 3 Department of Internal Medicine, Radboud University Medical Center Nijmegen, Nijmegen, the Netherlands; 4 Department of Online Learning and Instruction, Faculty of Educational Sciences, Open Universiteit, Heerlen, Netherlands; 5 Research on Learning and Education, Radboudumc Health Academy, Radboud University Medical Center, Nijmegen, Netherlands; 6 Department of Primary and Community care, Radboud University Medical Center Nijmegen, Nijmegen, Netherlands

**Keywords:** Clinical reasoning, Assessment, Medical education, Undergraduate education

## Abstract

Recently, a new digital clinical reasoning test (DCRT) was developed to evaluate students’ clinical-reasoning skills. Although an assessment tool may be soundly constructed, it may still prove inadequate in practice by failing to function as intended. Therefore, more insight is needed into the effects of the DCRT in practice. Individual semi-structured interviews and template analysis were used to collect and process qualitative data. The template, based on the interview guide, contained six themes: (1) DCRT itself, (2) test debriefing, (3) reflection, (4) practice/workplace, (5) DCRT versus practice and (6) ‘other’. Thirteen students were interviewed. The DCRT encourages students to engage more in formal education, self-study and workplace learning during their clerkships, particularly for those who received insufficient results. Although the faculty emphasizes the different purposes of the DCRT (*assessment of/as/for learning*), most students perceive the DCRT as an assessment ***of*** learning. This affects their motivation and the role they assign to it in their learning process. Although students appreciate the debriefing and reflection report for improvement, they struggle to fill the identified knowledge gaps due to the timing of receiving their results. Some students are supported by the DCRT in exhibiting lifelong learning behavior. This study has identified several ways in which the DCRT influences students’ learning practices in a way that can benefit their clinical-reasoning skills. Additionally, it stresses the importance of ensuring the alignment of theoretical principles with real-world practice, both in the development and utilization of assessment tools and their content. Further research is needed to investigate the long-term impact of the DCRT on young physicians’ working practice.

**Supplementary Information:**

The online version contains supplementary material available at 10.1007/s10459-023-10288-x.

## Introduction

Being an excellent clinical reasoner is one of the primary qualities of great physicians. Therefore, it is of the utmost importance that (future) physicians develop their clinical-reasoning skills throughout their careers (Jongbloed, 2002). This lifelong learning process is founded at the beginning of medical education and accelerates during clinical clerkships(Ruczynski et al., 2022). To support students in their learning process, institutions often include an assessment of clinical-reasoning skills in their curricula. This increasingly takes the form of an assessment program (Vleuten & Schuwirth, 2005) and can include various assessment formats, i.e. (in)formal, (non-)digital or practice-/education-based(Daniel et al., 2019; Lessing et al., 2020).

The selection and development of these assessment tools are usually driven by their intended purpose. Keeping these purposes in mind can help educators accomplish their goals with regards to their assessment. One of the most commonly used principles is *assessment of/as/for learning* (Winter, 2003). Assessment ***of*** learning has the longest and largest tradition within medical education. It focusses on testing for scores and gauging the intended outcomes of a medical curriculum rather than improving learning (Winter, 2003; Lockyer et al., 2017). These assessment tools are typically summative and positioned at the end of a semester, module or clerkship. Assessment ***as*** learning is defined as a situation where learning and assessment are intertwined to stimulate learners’ self-regulating learning behavior(Swan Sein et al., 2021). Examples include self-assessment, portfolios, reflection exercises and peer-assessment(Winter, 2003; Swan Sein et al., 2021). The last principle, assessment ***for*** learning, is mostly used within programmatic assessment. Within this principle, learners are centralized and assessment is seen as a tool to steer students’ learning by providing feedback on it(Kulasegaram & Rangachari, 2018). To that end, there has been a shift over the past years from assessment ***of*** learning towards assessment ***for*** learning(Winter, 2003; Lockyer et al., 2017). Despite having a well-constructed assessment tool, its practical inadequacy may arise when it fails to perform as originally intended. (Cook et al., 2015; Heeneman et al., 2015).

Recently, we developed a new digital clinical reasoning test (DCRT) to evaluate students’ clinical-reasoning skills. The DCRT is administered six times throughout the three-year master’s curriculum of medical school of the Radboud University Medical Center, each time at the end of a clerkship. The content of the DCRT is determined by the content of the clerkship students just completed. It uses ‘real world’ patient vignettes combined with different question types to help students develop their clinical-reasoning skills. This DCRT is the first tool of this type to combine six different specific question types that have all been studied separately for the assessment of clinical-reasoning skills(Beullens et al., 2005; Capaldi et al., 2015; Ber, 2003; Charlin et al., 2002).

Thus, to determine the usability of the DCRT, insight is needed into the effects of this assessment tool in practice. *Kane’s validity perspective* can help gather evidence on four different levels (scoring, generalization, extrapolation and implication) to determine the added value of an assessment tool for an assessment program(Cook et al., 2015; Kane, 2011). To this end, we interviewed students about their experiences with the DCRT to collect evidence for the Implication stage of Kane’s framework. The students’ answers should (1) provide more insight in the students’ perspective of developing clinical-reasoning skills both by assessment and in practice(Ruczynski et al., 2022), (2) clarify differences between assessment principles and how they work in practice, and (3) help us understand how integrating the clinical-reasoning practice into assessment can contribute to the clinical-reasoning learning process. Moreover, the results can be used to further develop the DCRT to strengthen its place in the curriculum and its assessment program. Our main research question to achieve these goals was: How does the DCRT influence medical students’ learning behavior, focused on clinical reasoning, during clinical clerkships?

## Methods

We used qualitative study methods to answer our research questions. This research is part of a larger project that gathers evidence alongside Kane’s validity framework. This research focuses on the Implications stage, and evidence gathering on other levels of the framework are currently ongoing and will not be presented here. Ethical approval was granted by the NVMO Ethical Review Board, case number 2021.6.6 (Netherlands).

### Setting

Medical education curricula in the Netherlands are organized in a Bachelor-Master-structure where each part takes three years to complete. The Master’s curriculum in Nijmegen alternates clinical clerkships with education at the university campus. Each clerkship is preceded and concluded with formal education to respectively prepare students for practice or evaluate and contemplate their clerkship. The education before clerkships (EBC) lasts between 1 and 4 weeks and the education after clerkships (EAC) 1–2 weeks. The first seven clinical clerkships follow each other in a fixed order in the first two years. In the final year, students choose their own specialty department for their last clinical clerkships and their scientific clerkship.

### The digital clinical reasoning test (DCRT)

The test that is developed and investigated in this research is the DCRT, which has replaced oral examinations in evaluating students’ clinical-reasoning skills in our curriculum. The DCRT is meant to be an assessment ***of, as*** and ***for*** learning. The test is administered at the medical faculty on the first day of EAC. A total of six DCRTs are taken during the first two years of the master’s curriculum. The content of each test is determined by the medical specialty in which they have just completed their clerkship. For example: the first clerkship is Internal Medicine, hence the DCRT after the first clerkship will contain questions about Internal Medicine. The question types used in this test are the same across all DCRTs: adapted script concordance test questions(Charlin et al., 2002), comprehensive integrative puzzles(Capaldi et al., 2015; Ber, 2003), extended matching questions(Beullens et al., 2005), multiple choice questions and short- and long-narrative answer questions. Students discuss the DCRT and their answers with a clinical expert in the week following the test.

Two weeks after completing the DCRT, students receive three scores: (1) their percentage score and (2) their percentile score of how they performed compared to their peers in both their own group and (3) their cohort (= 12 groups). Although there is no official pass or fail moment, these three scores together determine whether a student scores above or below a predefined cut-off point. In this study we will further refer to this as a ‘sufficient’ or an ‘insufficient’ result.

All students need to reflect on their test outcomes in a reflection paper. Students with an insufficient score need to reflect more broadly, subsequently write an improvement plan, set learning goals in this regard, and discuss these with their mentor. If students score insufficiently on two consecutive DCRTs, they will receive extra guidance and attention from their clinical supervisors during their next clerkship.

### Research population

Students who completed three DCRTs were invited to participate. A total number of 270 students were approached for participation via e-mail. Among the students who enrolled voluntarily, purposeful sampling was used in order to ensure that both students with sufficient and insufficient test outcomes were included from all years of the Master’s curriculum (Palinkas et al., 2015).

### Semi-structured individual interviews

We used individual interviews to provide a safe environment for students to discuss the DCRT and their personal experiences (Dicicco-Bloom & Crabtree, 2006) without pressure to provide socially desirable responses (Grossoehme, 2014). The interviews were semi-structured, using a set of predetermined topics that left ample room for other questions emerging from the dialogue (Dicicco-Bloom & Crabtree, 2006). Questions were categorized around six themes: (i) preparation, (ii) learning behavior, (iii) DCRT, (iv) test debriefing, (v) reflection and (vi) DCRT versus practice. The interview guide was iteratively altered as data collection proceeded and the last version can be found as an appendix.

### Data collection

The research group consists of three medical doctors (LR, MvdP, BS) with clinical experience in various departments (emergency medicine, primary and elderly care, internal medicine and clinical pharmacology), a cognitive psychologist (EC) and an educationalist with medical background (CF). Except one, all researchers are actively engaged in the medical curriculum as teachers (LR, MvdP, BS), curriculum developers (LR, MvdP, BS, CF), clinical supervisors (MvdP, BS), program director (MvdP) or as educationalist (CF). One researcher works as a teacher and curriculum developer in the educational science work field (EC).

This study was conducted between April and June 2022 at the Radboud University Medical Center in Nijmegen, The Netherlands. All interviews were conducted and transcribed by the head researcher (LR) and lasted between 20 and 45 min. LR does not engage with students in practice and is not involved in the assessment of DCRT results. Interviews were audio recorded and transcribed verbatim within two weeks after the interviews took place. The recordings were deleted afterwards. The names of the participants and any other traceable information was anonymized for confidentiality purposes. Saturation was reached when no new themes could be determined from the data and the research group determined that there was enough data to answer the research question(Varpio et al., 2017).

### Data analysis

Template analysis was used to process the data to ensure that necessary themes were incorporated in the analysis (King et al., 2004). The template was based on the interview guide and contained six themes: (1) DCRT itself, (2) test debriefing, (3) reflection, (4) practice/workplace, (5) DCRT versus practice and (6) ‘other’. LR and MvdP constructed a first template, which was further developed through group discussion (LR, MvdP, BS, CF) when everyone was familiarized with the data. After finalizing the template, all interviews were then coded by both the head researcher (LR) and one of the other researchers (MvdP, BS, CF). Subsequently, these codes were discussed in one-on-one sessions between the two coders until consensus was reached. Reports of these conversations were then discussed in a group session and used to start analyzation (LR, MvdP, BS, CF, EC). Analysis continued during the following two group sessions (LR, MvdP, BS, CF). Further modifications to the interpretations were made during the writing process, as well as through continuous discussions (LR, MvdP, BS, CF, EC).

### Writing process

Besides utilizing the services of a professional translation agency, ChatGPT was also employed to enhance the linguistic proficiency of this manuscript. ChatGPT did not partake in the analysis, nor did it generate any original content.

## Results

We interviewed 13 students: four of them completed four DCRTs, five students completed five DCRTS and four students completed six DCRTs. Their test results varied: five students had only sufficient results, five students received one insufficient result and three students had two or more insufficient results. First, we will provide the results on the students’ perceptions of the DCRT in general. Further results are categorized around the various iterative stages of the DCRT that students go through each clerkship: preparation, test, test debriefing, reflection, and practice. Quotes are used as a supplement to the data.

### The students’ perceptions of the DCRT in general

Students attach great significance to the first DCRT. The two explanations that are used most are not knowing what to expect and wanting to perform well. Over the course of the Master’s, the DCRT becomes less important to them. Some strongly believe that completing the DCRT is merely a requirement to obtain their medical degree. Arguments given by the students for this attenuated opinion of the DCRT include its placement in the EAC, their belief that the DCRT sometimes provides an inaccurate representation of their clinical-reasoning skills (see also ‘Test’ section), and that they consider practice as the golden standard for their clinical-reasoning learning process. Apart from these reasons, students do see and use this test to check if their current clinical-reasoning skills are on course to the expected level for their degree. They also use it to review content and to feed their confidence.

*And that it has given me a kind of confirmation that my knowledge is actually good. So that you can build more on that and become a little more confident and that you dare to ask a question more quickly. And that you don’t think… oh, this is a very basic question. But that you think … no, it’s not weird that I don’t know this.*

When students achieve an insufficient result, the DCRT regains some significance. This corresponds with the number of insufficient results. Although one insufficient result is usually not considered a disaster since it has hardly any formal consequences, it did instigate reflection and growth (see also ‘Reflection’ and ‘Practice’ sections). Two or more insufficient results do increase motivation to be more engaged, but they are also reasons for concern, lower self-confidence, and stress, as this means that they will be monitored extra during the next clerkship.

### Preparation

Students differ in the way they prepare for the DCRT. Some feel it was promoted to them as a test for which no preparation was needed. They assume that completing the clerkship is sufficient, while others carefully prepare themselves.

*It’s not about passing the test or not, but just to see where your areas of improvement are. So, I think it’s actually, kind of…um…a false result if you prepare. Because in real life you don’t prepare for every little thing. You go in with the knowledge you have. And I think that a clinical-reasoning test is an excellent opportunity to see how much knowledge you have.*

They unanimously see preparation as studying rather than practice. Although not necessarily recognized as such by students, elements of their practice can be labeled as preparation. They talk about independently preparing and seeing patients and talking to both supervisors and peers as strategies to enhance their clinical-reasoning skills.

*I just notice, for example, when I see patients or I’m chatting with a doctor about a patient that I’m very much thinking about history, physical examination, management. […] And that’s largely what you see in the test as well. So, during my internship I’m not thinking, ‘I’m doing this now, so I’m going to pass that test’. It’s more that because I do it so often on my clerkship that during the test I think ‘oh yes – this was so because I saw it then and then’.*

One strategy students use in preparation is to make lists of subjects they come across during their clerkship that they can study during a quieter moment of their workday. Apart from clinical practice, most students do not do anything special to prepare for the test. They feel they put more than enough time into their clerkship and want to protect their off-work time. Students who study outside of clinical practice mostly do this in the weekend between their clerkship and the DCRT. They study from books both the subjects they did not see in practice as well as the subjects they feel might come up in the DCRT. The university provides a list of topics for this purpose, which not all students know exists.

*Well, you know there’s always this open-ended question at the beginning [of the test]. You just know that somebody’s going to come into the Emergency Department (ED) or General Practice. So then, you can think, … okay, what would come into the ED or General Practice for Surgery or Neurology. […] There will always be a question about ‘What kind of diagnostic tests do you want?’ So, you must know what kind of diagnostic tests were brought up in that clerkship. […] That does help, when you’re prepared like that.*

*I do think that … suppose I don’t see certain things at all. We have a list of topics that I then peek into. Then I study only the things I really haven’t seen separately, because I think … otherwise I don’t know anything about them. And I hear that from some fellow students who do the same thing. That they study a little harder on other subjects because they don’t know anything about them.*

### Test

When asked about their performance on the DCRT compared to clinical practice, several different points emerge from students’ comments. First, students differ on the fact that the test provides feedback while they take it. Some find it helpful to know if they are on the right path. If they are not, they then correct their path and move on. However, others find it stressful to see they have already made a mistake and begin to worry about their result.

Secondly, students have different beliefs about the alignment of the DCRT with practice. Students who score poorly on the DCRT believe that this does not accurately reflect their performance in clinical practice, as they cannot use references during the test to aid their clinical-reasoning process. This difference is particularly evident in questions on (therapeutic) management. Still, they do feel that the way in which the test allows them to use clinical-reasoning steps reflects how they will do it in practice. They find that the steps are smaller in the DCRT compared to practice, where steps are sometimes simultaneously completed. They mention that the smaller steps force them to think more analytically about the problem, which ultimately helps them to do the same in practice.

*But then you are actually forced to think ‘what is actually the most essential thing to do’ or ‘what is really the first step you take, what should you really do’. And that is still good to think about sometimes, and to realize … oh yes, you really do* that *first, because that ultimately has the most effect on the course of the problem.*

Overall, students appreciate the DCRT for its broad spectrum of topics and question types. In contrast, most students also feel that the content does not align with clerkships at the university hospital since the DCRT focusses on general clinical-reasoning skills, while rare or complex cases are seen in academic hospitals. Some compensate by studying topics from the other clinical contexts prior to the test.

### Test debriefing

After completing the DCRT, students engage in a supervised debriefing. Experiences highly depend on the supervisor. Some merely display the answers and ask whether there are questions about them, while other teachers elaborate on all the steps to give insight into their own clinical-reasoning process. The latter is more appreciated by students, although both students and teachers struggle with the fact that there is ultimately one best answer. Teachers who fail to clearly explain the reasoning behind the right and wrong answers are unhelpful to students’ trust in the DCRT. Overall, the test debriefing is rated as insightful and helpful as a supplement to the test itself and the students’ clinical-reasoning learning process.

*[…] and that has to do with the fact that during the debriefing people very often say ‘yes, but this can be reasoned both ways. I understand why you said answer b with that explanation, but we only count answer c as correct’. And then I think sometimes… well… what exactly are we assessing here?*

### Reflection

Students must reflect on their test results. Students who have had only sufficient results see the reflection report as something mandatory and describe theirs as concise, saying they use the same text repeatedly. After an insufficient result, students reflect more extensively, seeking explanation in both internal and external factors.

*There were a number of reasons, in my opinion, why I didn’t pass the [test] for Pediatrics; among them, the fact that I had the clerkship at the [academic hospital] and that there are just very complex cases there. Common pediatric cases are not covered there very much. And I had missed the classes on the topics that came up a lot in the test. […] Besides that, I noticed that I needed to think a little more about problem management. […] That’s what I then focused on a little bit more, so that in the future I could answer [those questions] better.*

Most students experience difficulty reflecting properly, seeing as it is currently impossible to view their results in detail. Whether their reflection report is discussed with their mentor depends largely both on how the mentor appreciates the process and the student’s need to discuss it. Students are struggling with the urge to improve themselves on the one hand, and the formal position of the DCRT in the curriculum, on the other. They receive their results two weeks after completing the DCRT when they are already busy with their next clerkship. Only a few students mentioned intending to study during their next clerkship to compensate for their identified knowledge gaps from their previous clerkship in order to become better physicians.

*We were talking about that test and then my coach said, yes, it is indeed meant to show knowledge gaps of what do you not yet know so much about. But since it takes place after the clerkship, you don’t study after it, so to speak. You are then busy with the EAC and then with the EBC.*

*In my last DCRT there was also this syndrome, SOTOS syndrome. I had never heard of that, and afterwards I found out that my fellow students had not heard of that either. […] Well, then you just go and look that up. Yes, I do want to know what it was then. […] Well, it is asked on a test. So apparently, I am expected to know this, and I don’t. And then I’m just curious about it, I want to know. […] I don’t sit and study it at length because, indeed, you move on to the next clerkship. […] Because I think I want to become a general practitioner (GP). And then I think… I have to know something about that if I want to become a GP later.*

### Practice

Students look for ways to improve their learning, especially when they have received insufficient results. They put more effort into the EBCs, or they purchase a small notebook in which they collect information, questions, and subjects to explore further. These notes guide them in their studying in addition to making information easily accessible. Students who have had consistently sufficient results usually do not change their learning behavior, as they feel that what they are doing is already enough for them at this point.

*Um, no. I think those two things, distinguishing main and secondary issues and looking for the specific things that make you arrive at a certain working diagnosis. And really explicitly naming what you think about and then explicitly elaborating on your thinking steps and asking for feedback on that. Those are really the only things that changed as a result of this test – or, well, changed…in any case, I have gained these things from this test.*

*I partly think so because, for example, there are also questions about ‘what’s more or less likely’ or about certain diagnostics that you request. If I don’t know that, then I’ll make sure that I try to pay more attention to it during my next clerkship. Okay, why do they do that? Why do they choose this or why do they choose that? So that you know the reasoning behind it.*

## Discussion

This study has identified several ways in which the DCRT influences students’ learning practices in a way that can benefit their clinical-reasoning skills. Among other things, the DCRT encourages them to engage more in formal education as well as in workplace-learning during their clerkship. This is primarily the case for students who have received an insufficient result. Although the faculty emphasizes the different purposes of the DCRT, that is, an *assessment of/for/as learning*, students predominantly perceive the DCRT as an assessment ***of*** learning.

Our most interesting finding was the competing purposes of the DCRT when looking at the framework mentioned in the introduction, *assessment of/as/for learning*(Winter, 2003). The perception of students towards the DCRT indicated that most of them view it in a manner consistent with assessment ***of*** learning(Winter, 2003; Lockyer et al., 2017). This standpoint is supported by the contextual circumstances of the DCRT, such as its placement in the EACs and the semi-summative approach to the scoring. As a result, students’ motivation and the role they assign to the DCRT in their clinical-reasoning learning process are affected. Since assessment throughout the student’s lives has mostly been summative and set up for what they have previously learned (assessment ***of*** learning), it is not surprising they tend to treat the DCRT in the same way. More help is needed from institutions and educators at the practical end to facilitate the necessary transformation in students’ thoughts about assessment purposes in general and the DCRT’s goals specifically(Mezirow, 1997). At the same time, faculty should evaluate whether having three purposes for one assessment is desirable or necessary, as dual – or in this case, triple – purposing can hinder learners(Watling & Ginsburg, 2019; Gauthier, 2023).

Opposite to what is stated above, students do acknowledge that both the DCRT’s debriefing and reflection report facilitate improvement by offering valuable feedback and insights. This phenomenon is, in turn, linked to assessment ***for*** learning(Kulasegaram & Rangachari, 2018) and *lifelong learning*. Previous studies highlighting the importance of lifelong learning state that it is a desirable professional attitude when working in the everchanging healthcare system(Jongbloed, 2002). Therefore, fostering a positive attitude towards lifelong learning is in the interest of medical curricula(Murdoch-Eaton & Whittle, 2012). However, students struggle with giving their DCRT result a proper follow-up in terms of filling the identified knowledge gaps, since they receive their result after two weeks when they have already started the EBC of their next clerkship. This can inhibit the growth of lifelong learning behavior in students. Changing the DCRTs placement in the curriculum can offer a simple solution for this.

On the other hand, our results do indicate that part of the students are supported by the DCRT in developing lifelong learning behavior when it comes to shaping their learning activities in practice during their next clerkship. They formulate learning objectives that may pertain to specific aspects of the clinical-reasoning process (i.e. ‘I need to allocate more effort towards creating a treatment plan’) or more broadly relate to their learning behavior (i.e. ‘I need to take notes of unfamiliar topics encountered during practice and study them later in the day’). This highlights the DCRT’s ability to support *self-directed learning* among students within the clinical-reasoning learning process in clinical context(Houten-Schat et al., 2018; Vleuten & Schuwirth, 2019). This is a characteristic of assessment tools used for assessment ***for*** learning and stimulates students to develop behavior consistent with *lifelong learning*(Ricotta et al., 2022).

Furthermore, previous literature states that attitudes consistent with lifelong learning can be activated with practice-based education(Woezik et al., 2020). Our findings show that the way in which the DCRT allows students to utilize their clinical-reasoning skills is similar to how they would do it in practice. Therefore, it can be hypothesized that assessment tools incorporating practice into education could support lifelong learning as well.

Focusing on the clinical-reasoning learning process, our research revealed that students (un)consciously adapt their behavior in response to the DCRT. First, as they perceive the DCRT to be aligned with the way they would use their clinical-reasoning skills in practice, they also employ the test’s question types in practical settings. This shows that students learn for future practice by completing the DCRT, which can be seen as assessment ***as*** learning(Swan Sein et al., 2021): They undertake measures to aid their learning process and to ensure a deeper understanding of the subjects and processes, for example, by taking notes, thus bridging the gap between theory and practice.

Overall, one can be hesitant about what behavior change can be appointed to the DCRT. The structure of the DCRT and how its implemented invites certain behavior(Gibson, 1977). But looking at the DCRT, using different assessment principles, there are different types of behavior it can afford. Depending on the agency of the individual student, the level of autonomy and control someone feels in any situation(Reed, 1996), they treat the DCRT as an assessment ***of/as/for*** learning(Withagen et al., 2012). Further research could be done among young physicians to investigate which behavior that is supported by the DCRT has become a pertinent part of their working practice.

### Strengths and limitations

The inclusion of a diverse research population represents a significant strength of this study. The composition of the research group also contributed to a broad perspective and various angles during the lively discussions, thus limiting the possible bias due to hindsight bias or researcher bias. Additionally, the fact that a single moderator conducted all interviews is considered a strength because she can build upon previous interviews. However, limitations were identified in the specific context in which the research was conducted, as the behavior of Dutch students may differ from that of students in other regions of the world. Despite the attempt to create a safe and open context to minimalize this, there was a chance of social-desirability bias.

### Implications for practice and future research

The key takeaways from this study are that despite having a strong blueprint for an assessment tool, its implementation must be aligned with the theoretical purposes as well, as practice tends to hold greater influence than theory. Furthermore, the findings from this study can be utilized in broader reflective research on the DCRT or other assessment tools, for example by exploiting *Kane’s validity perspective* to determine the added value of an assessment tool for an assessment program(Cook et al., 2015; Kane, 2011).

## Conclusion

This study explores how the DCRT influences students’ clinical-reasoning learning practices. The DCRT encourages students to engage more in formal education, self-study and workplace learning during their clerkships, particularly for those who received insufficient results. Most students perceive the DCRT as an assessment ***of*** learning, affecting their motivation and the role they assign to it in their learning process. Although students appreciate the debriefing and reflection report for improvement, they struggle to fill the identified knowledge gaps due to the timing of receiving their results. Some students are supported by the DCRT in exhibiting lifelong learning behavior. This research stresses the importance of ensuring the alignment of theoretical principles with real-world practice, both in the development and utilization of assessment tools and their content,, to ensure that an assessment is correctly perceived. Further research is needed to investigate the long-term impact of the DCRT on young physicians’ working practices.

### Electronic supplementary material

Below is the link to the electronic supplementary material.

Supplementary Material 1

Supplementary Material 2
