# Supplementary material for: The influence of a digital clinical reasoning test on medical student learning behavior during clinical clerkships
Source: Adv Health Sci Educ Theory Pract. 2023 Oct 18;29(3):935–47. doi: 10.1007/s10459-023-10288-x (PMC11208212; doi:10.1007/s10459-023-10288-x)
Supplement: Supplementary file 1 — Supplementary Material 1 [file 10459_2023_10288_MOESM1_ESM.docx]

**Interviewguide**

Main research question: How does the DCRT influence medical students’ learning behavior, focused on clinical reasoning, during clinical clerkships?

**Research population:** undergraduate medical students from the Radboud University/Radboud University Medical Center who have completed a minimum of three DCRTs.

*Opening queastions*

- How far along are you in your Master’s?
- Which DCRTs have you completed?
- What were the results you have received?

*Main subjects*

| Subject | Topics |
| --- | --- |
| Preparation | - Practice - Formal education - Peers |
| Learning behavior | - Development of clinical-reasoning skills - Changes in behavior following the DCRT |
| DCRT itself | - Assessment strategy - Perceptions of the DCRT - Learning while making the DCRT |
| Test debriefing |  |
| Reflection report |  |
| DCRT vs practice | - Content - The way of using clinical-reasoning skills - Feedback |
