# Supplementary material for: The influence of a digital clinical reasoning test on medical student learning behavior during clinical clerkships
Source: Adv Health Sci Educ Theory Pract. 2023 Oct 18;29(3):935–47. doi: 10.1007/s10459-023-10288-x (PMC11208212; doi:10.1007/s10459-023-10288-x)
Supplement: Supplementary file 2 — Supplementary Material 2 [file 10459_2023_10288_MOESM2_ESM.pdf]

Sample questions

Multiple choice and adapted Script Concordance Test questions

Case
A 68-year-old man comes to the nephrology outpatient clinic. After having a blood test at the general practitioner's office due to diarrhea, an elevated creatinine level was found. He has had diarrhea for 5 weeks, progressively worsening in severity. He now has 10 to 15 bowel movements per day, in small amounts, loose, but without blood or mucus. He has no abdominal pain, nausea, or vomiting.
The patient mentions that he eats less, not because he feels nauseous but because he believes it has no effect due to the diarrhea. Additionally, he has been drinking little lately. Since last week, he has also had nighttime bowel movements, which disrupt his sleep. This prompted the patient to see the general practitioner.
The general practitioner performed a stool culture, but no pathogenic bacteria were found. Blood tests revealed acute kidney insufficiency, hence the referral to the hospital.
The patient has a urostomy and noticed that he has produced less urine during the past period. He has not seen blood in the urine. He sometimes appears somewhat confused. During the diarrhea, the patient continued taking his medications. He has lost three kilograms.

Medical history:
2016-06: TURT (Transurethral resection of bladder tumor), complicated by urinary tract infection and catheter-induced urinary retention. Pathology: urothelial cell carcinoma of the bladder.
2016-07: Cystectomy and creation of a Bricker bladder, pTisR0N1 (very limited lymph node involvement). Subsequently, poor wound healing, and it took six months for the wound to heal.

Medication:
Allopurinol tablet, 300 mg, oral, 1x daily
Clopidogrel tablet, 75 mg, oral, 1x daily
Digoxin tablet, 0.125 mg, oral, 1x daily
Metoprolol succinate tablet MGA 100 mg, oral, 1x daily
Pantoprazole tablet, 40 mg, oral, 1x daily
Paracetamol tablet, 1,000 mg, oral, 4x daily
Rosuvastatin tablet, 20 mg, oral, 1x daily
Furosemide 40mg
Spironolactone tablet, 25 mg, oral, 1x daily

There is acute renal failure.

Questions:

When examining the urine sediment, erythrocyte casts are found in the urine. Does this make a difference for the chance of an autoimmune condition?

1) Yes, that increases the chance.
2) Yes, that decreases the chance.
3) No, that does not affect the chance.

Protein is found in the urine. Does this make a difference for the chance of an infection?

1) Yes, that increases the chance.
2) Yes, that decreases the chance.
3) No, that does not affect the chance.

Distinction is made between prerenal, renal, and postrenal renal failure. A renal cause becomes more likely if there are abnormalities in:

1) An ultrasound of the kidneys.
2) Examining a urine sediment.
3) Measuring orthostasis.

Based on the case and the medications used, abnormalities in electrolyte balance are expected. Which electrolyte should be checked first due to the use of digoxin?

1) Calcium
2) Potassium
3) Magnesium
4) Sodium

It turns out that this patient has hyperkalemia: potassium 7.2 mmol/l. What is the first appropriate step in the treatment?

1) Administer intravenous calcium.
2) Initiate dialysis.
3) Administer intravenous magnesium.

Comprehensive Integrated Puzzle

Case

Mr. van B., 42 years old, is referred to the internist by his general practitioner (GP) due to long-standing joint pain. The pain is not daily but occurs often enough to have visited the GP multiple times. The GP did not find any clear abnormalities during the physical examination and prescribed NSAIDs. However, the complaints have not improved with this treatment. The most painful areas are the back, feet, right knee, and both wrists.

His medical history includes an appendectomy and a left inguinal hernia repair. Besides NSAIDs, he does not take any other medication. He drinks 2-3 beers during the weekends and smokes 3-5 cigarettes daily. He is in a committed relationship and has two children aged 6 and 8 years old. He works 36 hours a week in construction. His mother has hypertension and type 2 diabetes, and his father has experienced a hernia.

Question: The internist considers osteoarthritis, rheumatoid arthritis, axial spondylarthropathy, and gout. Complete the table with the most appropriate answers for the medical history and physical examination. One answer is not used each time.

**Diagnosis**	**History**	**Physical Examination**
Arthrosis		
Axial spondylarthropathy		
Gout		
Reumatoid arthritis		

<u>History:</u>

A. The complaints started in the wrists and fingers.
B. There is a burning sensation and pain during urination.
C. Use of thiazide diuretic.
D. Back pain.
E. Especially during work, there are many complaints.

<u>Physical Examination:</u>

A. Arthritis abnormalities (swan neck).
B. Erythema nodosum.
C. There are hard protrusions on the hands (Heberden's nodes).
D. There are small, hard, felt-like nodules under the skin (gouty tophi).
E. Uveitis.

Extended Matching Questions

Below are some common medications prescribed for hypertension. Choose the most appropriate medication/next step for each case. Answer options may be used more than once, and not all answer options need to be used. (1 point per question)

Answer options:
A. ACE inhibitor (lisinopril)
B. Aldosterone receptor antagonist (spironolactone)
C. Angiotensin receptor blocker (valsartan)
D. Beta-blocker (metoprolol)
E. Dihydropyridine calcium antagonist (amlodipine)
F. Digoxin
G. No medication adjustment
H. No pharmacological treatment/lifestyle advice
I. Loop diuretic (furosemide)
J. Methyldopa
K. Thiazide diuretic (chlorothalidone)

Question:
A 48-year-old woman known with chronic kidney insufficiency (MDRD 55 ml/min/1.73m2) with proteinuria and hypertension (BP 142/96 mmHg). What is the most appropriate medication/next step?

Question:
A 48-year-old woman known with diabetes mellitus without microalbuminuria and hypertension (BP 142/96 mmHg) already using an ACE inhibitor. What is the most appropriate medication/next step?

Question:
A 52-year-old man with atrial fibrillation and a ventricular response of 100 bpm and hypertension (BP 145/95 mmHg). What is the most appropriate medication/next step?

Short- and long-narrative answer questions

A 75-year-old man with a history of T3N0M+ prostate carcinoma (diagnosed at the age of 71) presents at the oncology outpatient clinic. In addition to systemic therapy, he underwent external radiotherapy to the lower back and SI joints a year ago. He reports having bloody diarrhea for the past 2 weeks. He does not have a fever, and the medical history is not contributory.

Question: Based on this case description, what is the most appropriate diagnosis? (1 point)

Question: Name three other diagnoses (different from the above question) that are included in the differential diagnosis for this case description? (3 points)

Question: For all four diagnoses (from the two questions above), provide a follow-up question that helps to better differentiate between the different diagnoses. (4 points)

Diagnose	Follow-up question

Question:

Name additional diagnostic tests for all four diagnoses to better differentiate between them and justify why these investigations help in the differentiation (8 points).

Diagnose	Diagnostic tests	Argument
